# A Novel Chimeric Molecule of Heparanase and Ig-Fc Enables Histochemical and Cytochemical Detection of *O*-sulfated Heparan Sulfate

**DOI:** 10.3390/ijms262311293

**Published:** 2025-11-22

**Authors:** Jia Shi, Momoko Nakamura, Ryoya Baba, Sojiro Arakawa, Arisa Yamaguchi, Tomonori Hariya, Rin Suzuki, Yu Inazuki, Katsuhiko Takahashi, Makoto Tsuiji, Teruaki Oku, Mayumi Komine, Momo Shimekake, Kyohei Higashi, Masao Nakamura, Kazuki Sasaki, Motowo Nakajima, Tatsuro Irimura, Nobuaki Higashi

**Affiliations:** 1 Department of Biochemistry, Hoshi University School of Pharmacy and Pharmaceutical Sciences, 2-4-41, Ebara, Shinagawa-ku, Tokyo 142-8501, Japanka-takahashi@hoshi.ac.jp (K.T.); 2 Department of Microbiology, Hoshi University School of Pharmacy and Pharmaceutical Sciences, 2-4-41, Ebara, Shinagawa-ku, Tokyo 142-8501, Japan; m-tsuiji@hoshi.ac.jp (M.T.); oku@hoshi.ac.jp (T.O.); 3 Department of Dermatology, Jichi Medical University, 3311-1 Yakushiji, Shimotsuke 329-0498, Japan; mkomine12@jichi.ac.jp; 4 Department of Clinical and Analytical Biochemistry, Faculty of Pharmaceutical Sciences, Tokyo University of Sciences, 6-3-1 Niijuku, Katsushika-ku, Tokyo 125-8585, Japanhigase@rs.tus.ac.jp (K.H.); 5 Department of Peptidomics, Sasaki Institute, 2-2, Kandasurugadai, Chiyoda-ku, Tokyo 101-0062, Japan; m-nakamura@po.kyoundo.jp (M.N.); ksasaki@ncc.go.jp (K.S.); 6 Department of Oncopeptidomics, Tochigi Cancer Center, 4-9-13, Yonan, Utsunomiya 320-0834, Japan; 7 SBI Pharmaceuticals Co., Ltd., 1-6-1, Roppongi, Minato-ku, Tokyo 106-6019, Japan; motnakaj@sbigroup.co.jp; 8 Division of Glycobiologics, Juntendo University Graduate School of Medicine, 2-1-1, Hongo, Bunkyo-ku, Tokyo 113-8421, Japan; t-irimura@juntendo.ac.jp; 9 Department of Breast Oncology, Juntendo University Graduate School of Medicine, 2-1-1, Hongo, Bunkyo-ku, Tokyo 113-8421, Japan

**Keywords:** atopic dermatitis skin, Fc-chimeric molecules, heparan sulfate, heparanase, heparin, inflammatory leukocytes, *O*-sulfation, syndecan

## Abstract

A chimeric protein of heparanase and Ig-Fc was designed as a novel tool to expand the detection of structurally heterogeneous heparan sulfate (HS) and related glycosaminoglycans. The whole mouse heparanase gene was combined with the gene segment encoding the mouse IgG1 hinge-Fc domain. A point mutation E335A was inserted to disable putative HS degradation activity. Chimeric proteins consisted of the latent form of the enzyme devoid of HS degradation activity. The chimeric proteins bound to heparin, *N*-desulfated heparin, and *O*-sulfated *N*-acetylheparosan. Their binding spectrum to glycosaminoglycans differed from that of anti-HS mAb 10E4. The chimeric proteins bound to Kato III and A549 cell lines. The binding was reduced by knocking down EXT1 gene expression. One of the chimeric proteins stained the epidermal cells in the hyperplastic spinous layer of inflamed atopic dermatitis skin and inflammatory cells in the dermis, which were not stained with mAb 10E4. The protein stained a polarized structure on the surface of monocytic U937 and THP1 cells. Similar polarized structures were observed with anti-syndecan-1 antibody staining. The chimeric protein and anti-syndecan-1 antibody precipitated similar sets of proteins that included syndecan-1 from the lysates of U937 cells. These novel chimeric proteins are useful to detect HS abundant in *O*-sulfation in histochemical, cytochemical, and biochemical studies.

## 1. Introduction

Glycosaminoglycans (GAGs) are negatively charged polysaccharides that are covalently attached to core proteins of proteoglycans. GAGs are structurally characterized based on the following two points: First, repeating consecutive disaccharide units compose the backbone of GAGs. Chondroitin sulfate (CS) and dermatan sulfate comprise *N*-acetylgalactosamine and glucuronic acid residues. Heparan sulfate (HS), heparin, and hyaluronic acid (HA) comprise *N*-acetylglucosamine and glucuronic acid residues. Keratan sulfate comprises *N*-acetylglucosamine and galactose. Second, except for HA, a significant portion of sugar residues in GAGs is enzymatically modified with sulfation and epimerization. These modifications confer structural microheterogeneity to the polysaccharide sequence [1]. GAGs play essential roles in many biological processes, such as cell adhesion, growth, motility, and differentiation. Their functions encompass a wide spectrum depending on their intra- or extracellular localizations. On the cell surface, they are receptors for virus and extracellular matrix proteins such as laminin and fibronectin. They also function as co-receptors of cytokines and chemokines. In the extracellular space, they are one of the main components of extracellular matrices. In intracellular granules of mast cells, they are intracellular scaffold materials enabling condensed packing of cationic granular molecules [2]. Some unique sulfation/epimerization patterns in GAGs permit specific binding to their target molecules, such as the pentasaccharide (AGA*IA: GlcN(NS,6S)-GlcA-GlcN(NS,3S)-IdoA(2S)-GlcN(NS,6S)) and its target protein, antithrombin III. Other oligosaccharides have their own target proteins, such as FGF-2, herpes simplex virus type 1 glycoprotein D, etc. [3,4,5].

Tools for the specific detection of subtle differences among structurally heterogeneous GAG molecules are anticipated to advance our knowledge about their functions. For this purpose, monoclonal antibodies that can detect specific GAG structures, such as anti-HS/heparin antibodies [6,7,8,9,10,11], an anti-CS-E antibody [12], and a GAG-binding scFv antibody using phage display systems [13], have been established. The binding specificities of these antibodies have been examined [14,15,16,17,18]. To further expand the library of GAG-binding molecules, proteins and peptides that tightly bind to sulfated GAGs such as HS have been utilized, such as basic FGF-2, cochlin, and NT4 peptides [19,20,21]. HA-binding protein is frequently utilized to detect and quantify HA [22]. Although these useful materials have the capacity to bind a variety of GAG structures, new detection tools that fully cover the diverse and heterogeneous structures in HS are further desired.

Heparanase (Hpse) has been initially identified as a mammalian endo-beta-glucuronidase that cleaves HS in basement membranes into 5 to 10 kDa fragments. Hpse facilitates tumor cell invasion and vascularization, which are critical events in cancer progression [23,24,25,26]. A latent Hpse of 65 kDa (human) and 60 kDa (mouse) [27] is processed into a mature and active form by elimination of an intervening peptide (Ser110-Gln157 in human). The Hpse active site contains two glutamic acid residues, Glu225 (human) and Glu217 (mouse), which act as a proton donor, and Glu343 (human) and Glu335 (mouse), which act as a nucleophile [28]. Because Hpse-mediated cleavage generates relatively long and biologically functional HS fragments of 5 to 10 kDa, the enzyme has been believed to recognize unique carbohydrate structures on the HS chain. Substrate recognition specificity of Hpse has been studied from the enzymatic degradation pattern of GAGs and their oligosaccharides with known saccharide sequences. A modified heparin-based study indicated that 2-*O*-sulfation on IdoA or GlcA is essential for recognition by Hpse, but not *N*-sulfation [29]. A tetra- or hexaoligosaccharide-based study indicated that a -GlcN(6S)-GlcA-GlcN(NS)- sequence with an additional sulfate group at +1 or +2 is preferable for the cleavage [30,31,32]. The highly sulfated domain of HS, known as the S (or NS)-domain, is relatively resistant to Hpse-mediated cleavage, which could potentially contribute to maintaining the functional integrity of HS even after the Hpse degradation [32,33,34]. In syndecan-4, the cleavage site was determined as GlcUA at the boundary between the NS/NA and the NS domain [34]. Hpse can cleave the internal sequence of pentasaccharide AG/A*IA in heparin and low molecular weight heparin, which may reduce the binding efficiency to antithrombin III [35,36]. It is likely that the cleavable GAG sequence is initially recognized by a local microstructure in the Hpse protein. Two heparin-binding domains (HBD-1 and HBD-2) have been identified [37]. These domains contain consecutive cationic amino acid residues, which are involved in the recognition of negatively charged GAGs mainly via electrostatic interaction. The optimal length of a GAG chain required to bridge the two domains is estimated as an octa- to decasaccharide [38,39]. Also, the Hpse molecule has a relatively longer cleft for recognizing the GAG chains in the structure, which is suitable for the fine recognition of diverse GAG structures. These discussions have been supported by the crystallographic study of the human active form Hpse [40], in which involvement of sulfated groups on the heparin backbone and the cationic amino acid residues in HBDs of Hpse was demonstrated. The latent form of Hpse also binds to HS and heparin [41,42,43,44], which is reasonable because the latent form fully maintains the above two HBDs. Deduced from the unique features of Hpse, a Hpse molecule lacking the enzymatic activity is applicable to recognize a long stretch of the HS/heparin chain.

In the present study, by focusing on the unique binding moiety of Hpse, we designed a novel divalent Hpse molecule expressed as an Fc-chimera protein (Hpse-Fc). To avoid possible GAG degradation, we preferably selected the latent form Hpse. Insertion of a point mutation E335A (mouse) in Hpse further minimized potential enzymatic activity. Hpse-Fc without or with the point mutation (Hpse(wt)-Fc and Hpse(mut)-Fc, respectively) were prepared and examined for the binding properties to immobilized carbohydrates, cells, and tissues. These properties were compared with the binding properties of an established anti-HS mAb 10E4 [6].

## 2. Results

### 2.1. Biochemical Characterization of Purified Hpse-Fc Chimeric Proteins

**Figure 1:**
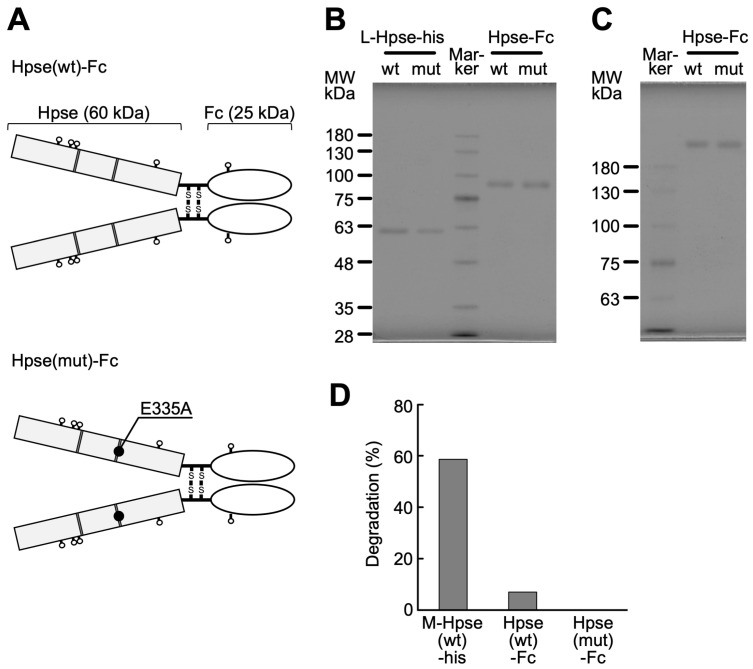
Biochemical characterization of Hpse-Fc chimeric proteins: (**A**) Structure of the Hpse-Fc proteins designed in the study. A pair of glutamic acid residues (dark gray) is required for the enzymatic activity. A point mutation E335A was inserted in the present study. (**B,C**) Elution profiles of the purified protein under reduced (**B**) and non-reduced (**C**) conditions in SDS-PAGE. In (**B**), recombinant mouse Hpse mimicking the latent form (L-Hpse (wt)-his [45]), L-Hpse(mut)-his (600 ng), and Hpse(wt)-Fc and Hpse(mut)-Fc (200 ng) were applied to a 9% separation gel. In (**C**), the same amounts of Hpse-Fc proteins were applied to a 6% separation gel. (**D**) HS degradation activity of recombinant mouse Hpse mimicking the mature form (M-Hpse(wt)-his [45]) and Hpse-Fc. These proteins were adjusted to a 100 ng/mL Hpse concentration in the enzymic assay solution and examined for their enzymatic activity.

Expression vectors of two types of Hpse-Fc chimeric proteins, one with wild-type mouse latent Hpse (Hpse(wt)-Fc) and another with a point mutation in E335A (Hpse(mut)-Fc), were constructed and expressed in 293T cells (Figure 1A). The chimeric proteins, released into the cell culture supernatant, were purified with a protein A sepharose column, using 1 M arginine solution buffered at pH 4.3 as an eluent. The two types of Hpse-Fc chimeric proteins were similarly purified as a single band of 85 kDa under reduced conditions (Figure 1B), and as a single band over 170 kDa under non-reducing conditions (Figure 1C). The chimeric proteins did not show any detectable degradation of fluorescently labeled HS up to 100 ng/mL (Figure 1D).

### 2.2. Hpse-Fc Chimeric Proteins Preferentially Bound to O-Sulfated GAGs

**Figure 2:**
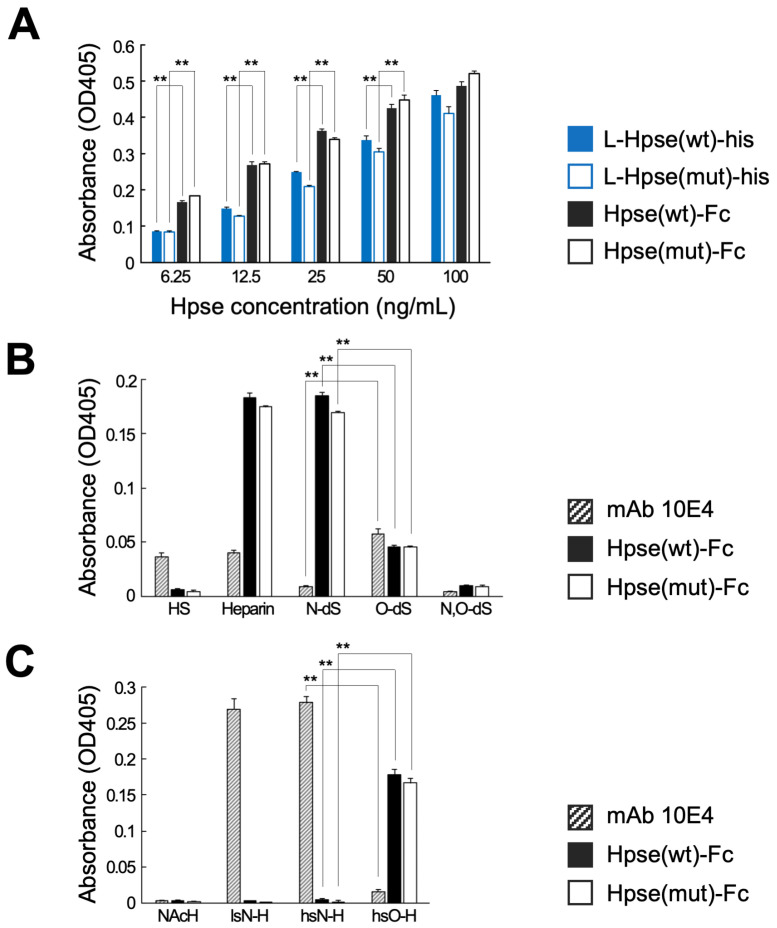
The Hpse-Fc chimeric protein bound to *O*-sulfated GAGs: (**A**) Binding properties of serially diluted L-Hpse-his and Hpse-Fc proteins (ng/mL as Hpse concentration) were examined using immobilized biotinylated heparin (5 µg/mL). (**B**) Binding properties of mAb 10E4 and Hpse-Fc proteins to biotinylated GAGs of HS, heparin, and its derivatives were examined. HS: heparan sulfate; Heparin: intact heparin; N-dS: *N*-desulfated heparin; O-dS: completely desulfated and *N*-resulfated heparin; N,O-dS: *N*- and *O*-desulfated heparin. (**C**) Binding properties of mAb 10E4 and Hpse-Fc proteins to biotinylated chemically sulfated polysaccharides were examined. NAcH: *N*-acetylheparosan; lsN-H: *N*-acetylheparosan, low-*N*-sulfated; hsN-H: *N*-acetylheparosan, high-*N*-sulfated; and hsO-H: *N*-acetylheparosan, *O*-sulfated. In (**B**,**C**), 10 µg/mL of diluted carbohydrates were added for immobilization. The concentration of mAb 10E4 and Hpse-Fc proteins in the binding experiments was 1 µg/mL and 50 ng/mL, respectively. Significant differences in binding were evaluated by two-tailed Student’s *t*-tests. **: *p* < 0.01.

The binding properties of Hpse-Fc and L-Hpse-his were examined. When the two types of molecules were adjusted as the concentration of Hpse, Hpse-Fc at a concentration from 6.25 to 50 µg/mL showed relatively higher binding to immobilized heparin than L-Hpse-his (Figure 2A). Hpse-Fc bound to intact heparin and *N*-desulfated heparin. The binding to completely desulfated and *N*-resulfated heparin, *N*- and *O*-desulfated heparin, or HS was reduced. Hpse-Fc did not bind to *N*-acetylheparosan but bound to *O*-sulfated *N*-acetylheparosan. The binding capacity to immobilized heparin was mostly comparable in Hpse(wt)-Fc and Hpse(mut)-Fc. mAb 10E4 had a different binding spectrum to a series of immobilized GAG libraries, i.e., it bound to heparin, HS, completely desulfated and *N*-resulfated heparin, and *N*-sulfated *N*-acetylheparosan. Hpse(wt)-Fc and Hpse(mut)-Fc showed relatively higher binding to *O*-sulfated glycans, whereas mAb 10E4 showed relatively higher binding to *N*-sulfated glycans (Figure 2B,C).

### 2.3. Hpse-Fc Chimeric Proteins Bound to Carcinoma Cell Lines via Cell Surface HS

**Figure 3:**
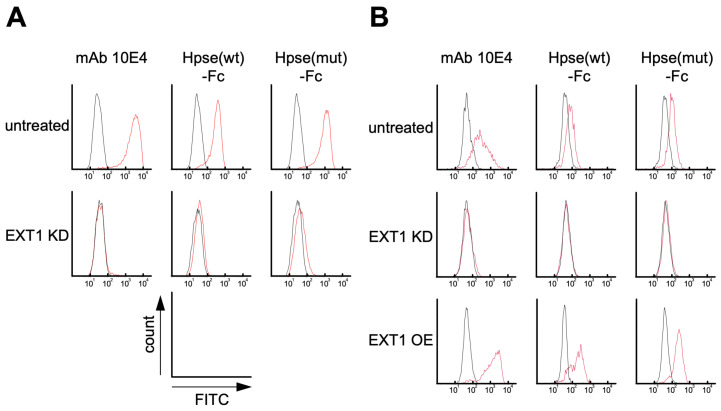
The Hpse-Fc chimeric protein bound to HS-expressing Kato III and A549 cells: (**A**) Kato III cells. Flow cytometry histograms showing the binding of mAb 10E4 and Hpse-Fc proteins to untreated and EXT1 KD cells. (**B**) A549 cells. Flow cytometry histograms showing the binding of mAb 10E4 and Hpse-Fc proteins to untreated, EXT1 KD, and EXT1 OE cells. Red lines: mAb 10E4 or Hpse-Fc; black lines: mouse IgG1 as negative control.

Binding of the Hpse-Fc chimeric proteins to Kato III and A549 cells was examined. Hpse-Fc and mAb 10E4 bound to the surface of these cells. Knockdown (KD) of the gene EXT1 by transfection with stealth RNAi greatly reduced the expression of cell surface HS, which was shown by reduced binding of mAb 10E4. The binding of Hpse-Fc chimeric proteins to EXT1 KD cells was substantially reduced, suggesting that the Hpse-Fc chimeric proteins bound to cell surface HS (Figure 3A,B). The EXT1 transgene was overexpressed (OE) in the A549 cells using a lentivirus system. The binding of mAb 10E4 to the A549-EXT1 OE cells, expressed as median fluorescence intensity (MFI) ratio, was significantly higher than the binding to untreated A549 cells, 35.1 ± 12.0-fold vs. 7.04 ± 1.75-fold (n = 3, *p* = 0.016), respectively. Moderately increased binding was present after staining with Hpse-Fc. The increase in the MFI ratio of Hpse(wt)-Fc was 3.32 ± 1.71-fold with A549-EXT1 OE vs. 2.29 ± 0.41-fold with untreated A549, and that of Hpse(mut)-Fc was 4.56 ± 1.97-fold with A549-EXT1 OE vs. 2.98 ± 0.95-fold with untreated A549 (n = 3) (Figure 3B).

### 2.4. Hpse-Fc Chimeric Proteins Bound to Epidermal Cells and Dermal Inflammatory Cells in Atopic Dermatitis Skin

**Figure 4:**
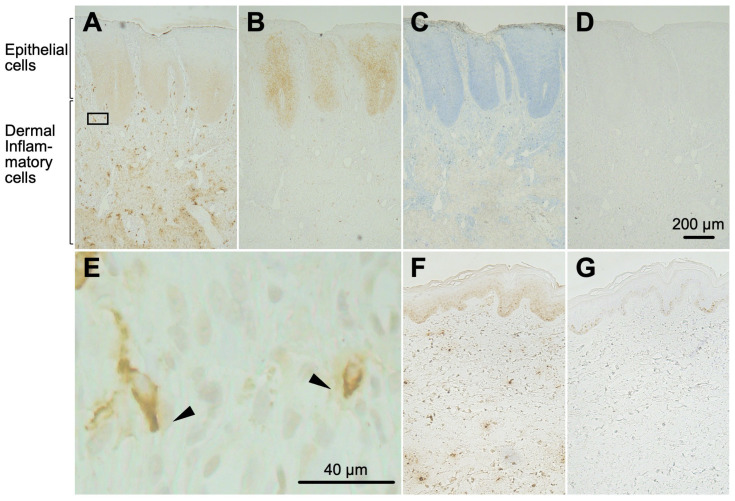
The Hpse(mut)-Fc chimeric protein detected epithelial cells and dermal inflammatory cells in atopic dermatitis skin. Thin sections (3 µm) of inflamed atopic dermatitis skin were stained with Hpse(mut)-Fc (**A**), mAb 10E4 (**B**), toluidine blue (**C**), or mouse IgG1 as a control (**D**). The indicated area in panel (**A**) is shown enlarged in (**E**), in which the Hpse(mut)-Fc-dependent binding site appears polarized in the inflammatory cells (arrowheads). Thin sections (3 µm) of healthy skin were similarly stained with Hpse(mut)-Fc (**F**) and mAb 10E4 (**G**). Bar: 200 µm (**A–D**,**F**,**G**); 40 µm (**E**).

Histochemical binding of a Hpse(mut)-Fc chimeric protein to sectioned skin specimens from atopic dermatitis patients was examined. Hpse(mut)-Fc moderately stained the hyperplastic spinous layer in the epithelium, and strongly stained a small portion of inflammatory cells in the dermis (Figure 4A). mAb 10E4 also stained the spinous layer in a similar way; however, this was not the case with dermal inflammatory cells (Figure 4B). The cells in the dermis were not detected with toluidine blue staining (Figure 4C). Mouse IgG1, as a negative control, did not confer any positive staining (Figure 4D). In some of the dermal inflammatory cells, the Hpse(mut)-Fc staining showed a polarized distribution (Figure 4E). In healthy skin sections, nuclei of the cells in the spinous layer showed dull staining, while the dermal cells were not stained (Figure 4F,G).

### 2.5. Hpse-Fc Chimeric Proteins Bound to Cell Surface Syndecan-1 in Monocytic U937 Cells

**Figure 5:**
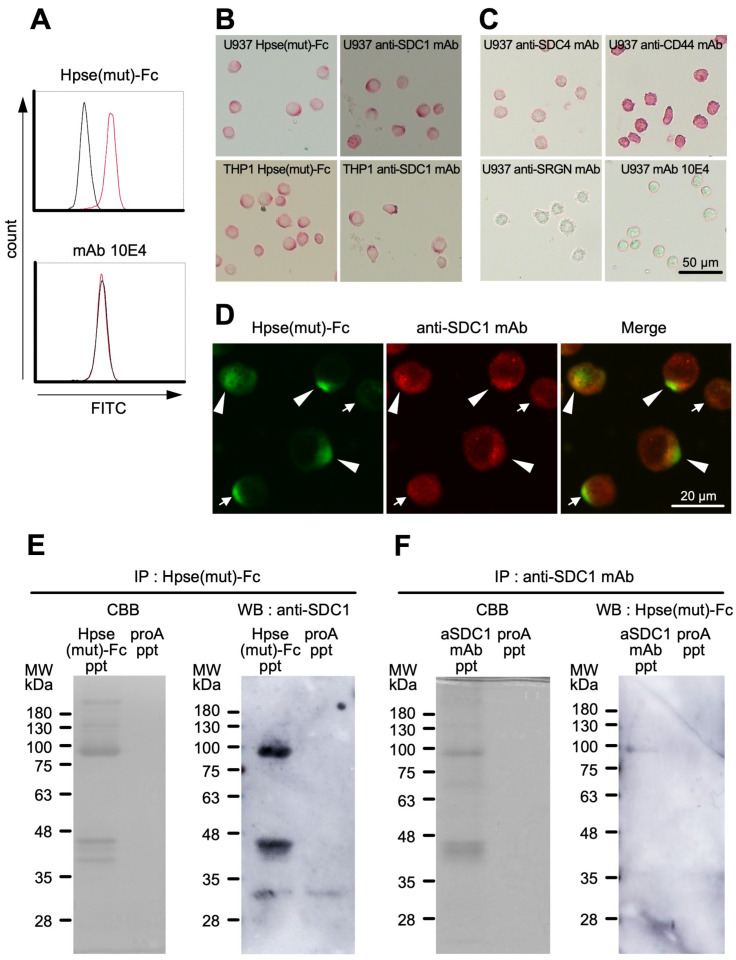
Hpse(mut)-Fc bound to PMA-treated U937 and THP1 cells: (**A**) The U937 cells were treated with 50 nM PMA for 48 h, fixed with 4% paraformaldehyde in suspension, and stained with Hpse(mut)-Fc or mAb 10E4. Flow cytometric histograms are shown. Red lines: Hpse(mut)-Fc or mAb 10E4; black lines: mouse IgG1 as negative control. (**B**) The U937 and THP1 cells were treated with 50 nM PMA for 48 h, adhered to glass slides for 2 h, fixed with 4% paraformaldehyde, and stained with Hpse(mut)-Fc and anti-syndecan-1 (SDC1) antibody. The binding was visualized using alkaline phosphatase. (**C**) The PMA-treated U937 cells were stained with mAb 10E4, and antibodies to putative HS-core proteins, i.e., syndecan-4 (SDC4), serglycin (SRGN), and CD44. (**D**) The binding of Hpse(mut)-Fc was visualized using rat anti-muHpse mAb (RIO1) and Alexa 488-conjugated goat anti-rat IgG (green). The binding of anti-SDC1 mAb was visualized using biotinylated goat anti-mouse IgG F(ab’)2 fragment and streptavidin-Alexa 568 (red). (**E**) The lysate of the adherent U937 cells treated with 50 nM PMA was treated with Hpse(mut)-Fc-conjugated protein A sepharose or unconjugated protein A sepharose. The bound fraction (Hpse(mut)-Fc ppt and proA ppt, respectively) was subjected to SDS-PAGE under non-reducing conditions and stained with CBB (**left**). The blotted membrane was stained with the anti-SDC1 antibody (**right**). (**F**) U937 lysate was treated with the anti-SDC1 antibody-conjugated protein A sepharose or unconjugated protein A sepharose. The bound fraction (aSDC1 ppt and proA ppt, respectively) was subjected to SDS-PAGE under non-reducing conditions and stained with CBB (**left**). The blotted membrane was stained with Hpse(mut)-Fc (**right**).

Binding of Hpse(mut)-Fc to inflammatory leukocytes was further examined using monocytic U937 and THP1 cells differentiated with PMA for 48 h [46]. The binding of Hpse(mut)-Fc to fixed U937 cells was detected by flow cytometry (Figure 5A). In immunocytochemistry, the staining of Hpse(mut)-Fc visualized polarized structures on the surface of the adherent U937 and THP1 cells (Figure 5B, left panels). Antibodies to a panel of putative HS-core proteins were used to test whether similar polarized staining was observable (Figure 5B,C). Anti-syndecan-1 (SDC1) mAb stained polarized structures on the cells (Figure 5B, right panels). Anti-syndecan-4 (SDC4) mAb stained the entire surface of the U937 cells weakly. Anti-CD44 mAb stained the entire body of the U937 cells strongly. Anti-serglycin (SRGN) and mAb 10E4 did not confer any positive signal (Figure 5C). Colocalization of Hpse(mut)-Fc binding site and SDC1 was further examined. The stained regions with Hpse(mut)-Fc and anti-SDC1 mAb partially overlapped (Figure 5D). Putative Hpse(mut)-Fc binding molecules in the lysate of the U937 cells were prepared using protein A sepharose-dependent precipitation. Coomassie Brilliant Blue (CBB) staining showed a dense band eluted around 90 kDa, which corresponded to Hpse(mut)-Fc itself. The precipitates of Hpse(mut)-Fc and the anti-SDC1 antibody showed a similar elution pattern in SDS-PAGE. SDC1 was detected in the precipitates of Hpse(mut)-Fc at apparent molecular weights of 95 kDa and 44 kDa. Similarly, Hpse(mut)-Fc binding molecules were detected in the precipitates of the anti-SDC1 antibody at an apparent molecular weight of 95 kDa, strongly suggesting that Hpse(mut)-Fc can bind to 95 kDa SDC1 (Figure 5E,F).

**Figure 6:**
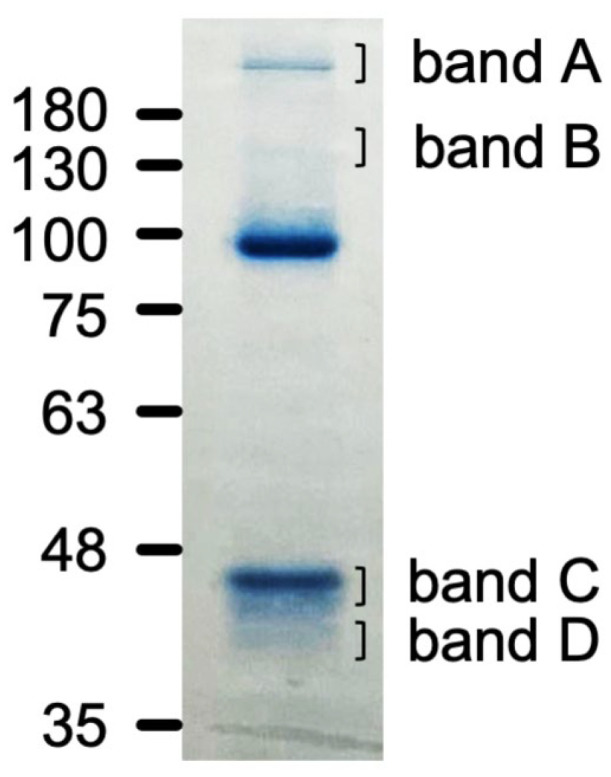
SDS-PAGE separation of the Hpse(mut)-Fc precipitates. The four bands in the panel were excised and subjected to in-gel digestion with trypsin for mass spectrometric identification.

Molecules interacting with Hpse(mut)-Fc were further analyzed using gel electrophoresis. CBB-stained bands had a molecular weight of over 180 kDa (band A), 130 kDa (band B), 44 kDa (band C), and 39 kDa (band D) (Figure 6). Peptide fragments derived from these bands were analyzed by liquid chromatography/mass spectrometry. Spectrin, actin-binding proteins such as alpha-actinin-4, and others were identified from band A and band B (Table 1a,b). Cytoplasmic actin was mainly identified from band C and band D (Table 1c,d and Table S1).

## 3. Discussion

In the present study, we prepared a novel tool to distinctly detect highly sulfated GAG structures. The Hpse-Fc chimeric molecules bound to immobilized GAGs (Figure 2). Judging from the binding spectrum, it is reasonable to suggest that Hpse-Fc prefers a highly sulfated structure with abundant *O*-sulfation, which was different from the binding spectrum of mAb 10E4. Hpse-Fc bound to GAGs expressed on the cell surface (Figure 3). Knockdown of the EXT1 gene greatly reduced the binding intensity, strongly suggesting that the target for the binding was HS. Hpse-Fc bound to GAGs in inflamed atopic dermatitis skin sections and in differentiated monocytic cell lines (Figure 4 and Figure 5). SDC1 was identified as a binding molecule in the lysate of the PMA-treated U937 cells (Figure 5). Taken together, these results can propose Hpse-Fc as a novel tool to detect highly sulfated GAGs in cells and tissues. Of note, a part of the sulfated structure was detectable with the present Hpse-Fc but not with mAb 10E4. Considering the applicability of Hpse-Fc for histological staining, it is a promising tool for expanding the spectrum of detectable HS/heparin microstructures.

A previous study examined the influence of environmental pH on Hpse enzymatic activity, showing that Hpse maintained its biological activity at pH 4 [25]. It is expected that the Hpse structure was intact under the mildly acidic conditions used in this study. To avoid any denaturation of Hpse under acidic conditions, the chimeric molecules were eluted at pH 4, which included a high concentration of arginine [47]. At a lower concentration, Hpse-Fc showed relatively higher binding to immobilized heparin than L-Hpse-his (Figure 2A), which suggests that Hpse in the Hpse-Fc molecules maintains its binding capacity.

Hpse-Fc bound to highly sulfated heparin in the panels of heparin derivatives (Figure 2B) and synthetic sulfated polysaccharides (Figure 2C). The binding spectrum of Hpse-Fc was different from that of mAb 10E4. It has been reported that binding of mAb 10E4 was greatly affected by the state of the GlcNH_3_^+^ amino group in HS, and by whether the N is unsubstituted, acetylated, or sulfated. A study suggested the relevance of unsubstituted NH_3_^+^ for the binding [16,17], while another study suggested that mixed *N*-sulfated/*N*-acetylated epitopes are required for the binding [18]. Involvement of *O*-sulfation in the binding of mAb 10E4 remains controversial [18,48,49]. In our study, mAb 10E4 strongly bound to completely desulfated and *N*-resulfated heparin and *N*-sulfated *N*-acetylheparosan; both permit the presence of mixed *N*-sulfated/*N*-acetylated epitopes. Relevance of the *N*-unsubstituted GlcNH_3_^+^ epitope was not clear, as it is not likely that *N*-sulfated *N*-acetylheparosan contains the epitope. It is more likely that *N*-sulfated/*N*-acetylated epitopes are relevant for the binding and that *O*-sulfation has a minimal effect on the binding. On the other hand, the binding of Hpse-Fc to heparin was not influenced by *N*-desulfation, as Hpse-Fc bound to *N*-desulfated heparin. The restored binding after *O*-sulfation of *N*-acetylheparosan suggested that *O*-sulfation greatly influences the binding of Hpse-Fc. Hpse potentially binds to GAG molecules other than HS or heparin, such as CS-E and sulfated HA carrying solely *O*-sulfation [50,51]. In this sense, Hpse-Fc is expected to have a binding spectrum that is different from mAb 10E4. Crystallography of mature Hpse with a heparin tetrasaccharide (dp4) suggested that a series of *N*- and *O*-sulfates on (−2) and (+1) carbohydrate residues are likely involved in the recognition [40]. Although information on interaction of latent Hpse with GAGs is limited, a similar interaction may occur with Hpse-Fc containing the latent form of Hpse. Hpse-Fc did not bind to immobilized HS in the experiment, possibly because the latent form of Hpse in the chimeric molecules had relatively low affinity to HS, or some of the carboxyl groups required for the binding were modified during the biotinylation reaction.

We examined whether Hpse-Fc can bind to HS on the cell surface. Both Hpse-Fc and mAb 10E4 bound to the cell surface of the Kato III and A549 cells, which was significantly reduced in the cells transfected with siRNA targeted to the EXT1 gene. The binding of mAb 10E4 to EXT1-overexpressing A549 cells was around 5-fold higher than the binding to untreated A549 cells, deduced from the increase in MFI values (35.1-fold vs. 7.04-fold), whereas this was not the case with the binding of Hpse(wt)-Fc (3.32-fold vs. 2.29-fold) or Hpse(mut)-Fc (4.56-fold vs. 2.98-fold). mAb 10E4 and Hpse-Fc commonly bind to HS, but it is possible that they may recognize different structures on the same HS chain [52].

HS is expressed throughout the body, while the distribution of various GAG structures has not been fully examined in histochemical studies. Using the novel HS-binding molecule Hpse(mut)-Fc, two characteristic staining patterns were apparent, i.e., a dull staining in the hyperplastic spinous layer of the epidermis, and a dense staining of a small portion of inflammatory cells in the dermis of atopic dermatitis skin (Figure 4). The positive signal in the spinous layer in the epithelium could be due to SDC4 expression in the region, as reported previously [53]. GAGs in the epithelium were similarly detected by Hpse(mut)-Fc and mAb 10E4, suggesting that the HS carries structures allowing it to bind to both Hpse(mut)-Fc and mAb 10E4. Dense staining in the inflammatory cells in the dermis was shown with Hpse(mut)-Fc, but such cells were not detected with mAb 10E4 or with toluidine blue staining. It is likely that cells other than connective tissue-type mast cells were stained with Hpse(mut)-Fc. The staining signal was localized in a restricted orientation in some of the inflammatory cells (arrowheads in Figure 4E), suggesting that the cells were polarized and potentially migrating in the tissues.

To further examine the binding of Hpse(mut)-Fc in leukocytes, differentiated U937 and THP1 cells were examined as a model of inflammatory leukocytes [46]. Hpse(mut)-Fc but not mAb 10E4 was able to bind to the U937 cells by flow cytometry or by immunocytochemistry. The regions in the U937 cells that were stained with Hpse(mut)-Fc and anti-SDC1 mAb partially overlapped. Immunoprecipitation experiments showed that Hpse(mut)-Fc and the anti-SDC1 antibody share similar sets of proteins for interaction.

Mass spectrometry analysis identified multiple cytoskeletal and cytoskeleton-associated proteins, including spectrins, alpha-actinin, filamin, and myosin, as co-precipitated with Hpse(mut)-Fc (Table 1). These findings suggest that Hpse(mut)-Fc interacts with the cytoskeletal protein network via SDC1, thereby linking extracellular signals to intracellular structural organization. This interaction may contribute to the polarized distribution of SDC1 and associated GAGs on the leukocyte membrane. Some of the identified proteins possibly interact with Hpse-Fc via non-specific binding. Addition of heparin-containing solution to the Hpse(mut)-Fc-conjugated protein A sepharose may preferentially elute proteins with carbohydrate-dependent binding.

Hpse(mut)-Fc can precipitate SDC1 of 95 kDa and 44 kDa; however, only 95 kDa SDC1 was detected by the binding of Hpse(mut)-Fc. It is feasible that the 95 kDa SDC1 was modified with HS, however, the 44 kDa SDC1 was not. As shown in a previous study, enzymatically active Hpse was expressed on the cell surface of U937 cells, which facilitates cellular invasion through basement membrane-like extracellular matrix [41]. The enzyme can be active to cleave the cell surface HS of SDC1, which may generate the 44 kDa form that does not bind to Hpse(mut)-Fc. This form likely consists of a core protein and an attached CS chain. A recent study suggests Hpse-mediated cleavage of HS on the cell surface of monocytes greatly accelerates the migratory capacity; therefore, the cleavage is physiologically significant [54]. The Hpse-mediated deglycanation of SDC1 may also accelerate subsequent shedding of SDC1 mediated by proteases such as MMPs and ADAM17 [55]. The role of Hpse-(mut)-Fc-binding HS in SDC1 shedding remains to be studied in the future.

In conclusion, the present study designed novel Hpse-Fc chimeric molecules aiming to detect highly sulfated GAG structures. These new molecules had a unique GAG binding spectrum different from a known anti-HS mAb 10E4. Using Hpse(mut)-Fc as a detection molecule, we newly detected Hpse(mut)-Fc-positive inflammatory cells in the dermis of atopic dermatitis skin and polarized HS-like structures, which were distributed together with SDC1 on the surface of the adherent U937 and THP1 cells. These findings have implications for the novel application of Hpse-Fc to detect highly sulfated glycans using histochemical, cytochemical, and biochemical methods.

## 4. Materials and Methods

### 4.1. Reagents and Cells

Triton X-100, bovine serum albumin (BSA) for cell culture, and phorbol myristate acetate (PMA) were purchased from Sigma (St. Louis, MO, USA); Tween-20 from Wako Pure Chemical (Tokyo, Japan); RPMI1640, DMEM and CHAPS from Nacalai tesque (Kyoto, Japan); normal goat serum from Japan Laboratory Animals, Inc. (Tokyo, Japan). The following antibodies were used: anti-HS (clone F58-10E4) from Amsbio (Madrid, Spain), anti- SDC1 mAb from Biolegend (DL101), anti-SDC1 antibody from Cell Signaling Technology (D4Y7H), anti-syndecan-4 (SDC4) mAb (5G9) and anti-serglycin (C-11) mAb from Santa Cruz Biotechnology, anti-CD44 mAb (IM1219U) from Beckman Coulter, anti-mouse IgM, HRP-conjugated goat anti-mouse and rabbit IgG(H+L), biotin-conjugated goat anti-mouse IgG F(ab’)_2_ fragment from Zymed (South San Francisco, CA, USA), non-labeled streptavidin from Fujifilm-Wako (Osaka, Japan), streptavidin labeled with alkaline phosphatase or FITC from Zymed, and Alexa 488- and Alexa 568-labeled streptavidin from Invitrogen (Carlsbad, CA, USA). Rabbit anti-muHpse antiserum and 6×His-tagged recombinant Hpse (L- and M-Hpse-his) were prepared as stated elsewhere [45].

### 4.2. Design and Preparation of Chimeric Proteins

The gene of mouse IgG1-Fc was inserted into an established pcDNA3.1(-) hygro-muHpse using the Gibson assembly system (New England Biolabs) following the manufacturer’s protocol. The pcDNA3.1(-) hygro-muHpse was digested with Hind III. The mIgG1 heavy chain gene derived from C57BL/6 was amplified using the primers as below.

Sense: aaaatcgctgcttgtataGGAGGGGGTGGATCAggttgtaagccttgcatatgtacag

Antisense: gatcagcggtttaaacttAAGCTTgtaggtgtcagagtcctgtaggac

The IgG1-Fc included hinge regions that allow dimerization of the chimeric proteins to form the Hpse-Fc protein. A set of primers was used for insertion of a point mutation at E335A as described elsewhere [45]. 293T cells were transfected with the vector encoding the chimeric proteins using lipofectamine 3000 (Invitrogen). After 48 h incubation, the cells were cultured with DMEM-10% FCS containing 300 µg/mL Hygromycin B for selection. The living cells were cloned and further screened based on higher production of Hpse-Fc in the culture supernatant. For collecting the supernatants, the semi-confluent cloned cells were washed with DMEM and cultured in PeproGrow HEK293 animal-free chemically defined HEK293 medium (Peprotech, Rocky Hill, NJ, USA), HE100 medium (Gmep Inc., Kurume, Japan), or ExCell 293 serum-free medium for HEK293 cells (14571C, Sigma-Aldrich, Burlington, MA, USA) for 4~7 days under adherent conditions.

### 4.3. Purification of Chimeric Proteins

The supernatant was diluted with an equal volume of TBS (20 mM Tris-HCl, 150 mM NaCl (pH 7.62)) and applied to a protein A sepharose column (Cytiva, Uppsala, Sweden). After washing with TBS, the column was washed with 20 mM Tris-HCl buffer containing 1.5 M NaCl to eliminate heparin and other proteins as potential contaminants in the cell-derived supernatant. Then the chimeric protein was eluted with 1 M arginine-HCl (pH 4.3), where the arginine was utilized to avoid acid-induced denaturation of Hpse. The eluate was quickly neutralized with 1 M Tris-HCl buffer (pH 9.0) and dialyzed against PBS to eliminate excessive arginine. The molecular weight of the Hpse-Fc chimeric molecules was estimated with conventional SDS-PAGE.

### 4.4. Heparan Sulfate Degradation Activity

HS degradation was detected as described elsewhere using Superdex 75 increase (5/150 GL, Cytiva) [50]. The magnitude of the degradation was quantified from HPLC chromatograms by measuring an area of 0.25 < Kav < 1 of the elution divided by the total elution area and indicated as degradation (%).

### 4.5. Binding Experiments to Immobilized GAGs

Heparin (from porcine intestinal mucosa, H7005, approx. 15 kDa) was purchased from Sigma. Biotinylated HS was kindly provided by Dr. Shuji Mizumoto (Meijo University). *N*-acetylheparosan (m.w. 35,318; NAcH); *N*-acetylheparosan, high-*N*-sulfated (*N*-sulfation ratio: 81%; hsN-H); *N*-acetylheparosan, low-*N*-sulfated (*N*-sulfation ratio: 39%; lsN-H); and *N*-acetylheparosan, *O*-sulfated (sulfation ratio: 3.83 per one disaccharide unit, m.w.: 34,872; hsO-H) were kindly provided by Tokyo Chemical Industry Co., Ltd. (TCI, Tokyo, Japan). Desulfation, acetylation, and resulfation of heparin were performed as described elsewhere [56]. In the present study, *N*- and *O*-desulfated heparin (N,O-dS), completely desulfated and *N*-resulfated heparin (O-dS), and *N*-desulfated heparin (N-dS), were used. The GAGs were biotinylated at their carboxyl groups using combined treatment with 1-ethyl-3-(3ʹ-dimethylaminopropyl)carbodiimide (TCI, D1601) and biotin-PEG4-hydrazide (TCI, B5578).

For immobilization of GAGs, streptavidin solution (5 µg/mL, 15 µL) was added to a half-size ELISA plate (Greiner 675061). After blocking with 1% BSA solution, the biotinylated GAGs (5 to 10 µg/mL, 20 µL) were added. After washing, mAb 10E4 or Hpse-Fc chimeric proteins were added to the wells and incubated for 1 h. The binding to these GAGs was detected using a horseradish peroxidase (HRP)-conjugated goat anti-mouse IgG (H+L) antibody (Zymed) and measured by coloration of 2,2′-azino-bis [3-ethylbenzothiazoline-6-sulfonic acid] (ABTS). For measuring the binding of anti-HS mAb (10E4, Amsbio), HRP-conjugated goat anti-mouse IgM (Zymed) was used for the detection.

### 4.6. Detection of Cell Surface Antigens Using Flow Cytometry

The Kato III cells (ATCC) were maintained in RPMI1640 with 10% FCS, and the A549 cells (ATCC) were maintained in DMEM with 10% FCS. The EXT1 gene encodes a glycosyltransferase required for HS biosynthesis [57]. The EXT1 gene was expressed in the A549 cells using a lentivirus expression system as described elsewhere [43]. In some experiments, Stealth RNAi^®^ (HSS103435) was used for suppression of EXT1 gene expression. For flow cytometric analysis, the Kato III, A549, and A549-EXT1 cells were harvested with 0.02% EDTA in PBS for 1 h, and suspended in FACS buffer (0.1% BSA, 0.01% NaN_3_ in PBS). After blocking with 2% normal goat serum, 3% BSA, 0.01% NaN_3_ in PBS, the cells were treated with mAb 10E4 (5 µg/mL) or Hpse-Fc proteins (5 µg/mL) as primary Ab, further stained with a secondary Ab (FITC-conjugated goat anti-mouse IgM (Biosource) or FITC-conjugated goat anti-mouse IgG (H+L) (Zymed)), and analyzed using FACS Verse (BD).

### 4.7. Histochemical Detection of Hpse(mut)-Fc Binding Sites in Human Skin

The histochemical study was approved by the Research Ethics Committee of Hoshi University School of Pharmacy and Pharmaceutical Sciences (No. 30-038).

Thin sections (3 µm) of human atopic dermatitis skin specimens were deparaffinized and stained with Hpse(mut)-Fc or mAb 10E4 (0.85 µg/mL). After treatment with the primary antibody, the sections were treated with biotinylated secondary antibody and Vectastain ABC kit (PK-4002). The staining was visualized with diaminobenzidine hydrochloride (Nacalai Tesque, Kyoto, Japan).

### 4.8. Cytochemical Detection of Hpse(mut)-Fc Binding Molecules in Monocytic U937 and THP1 Cells

The U937 and THP1 cells (ATCC) were treated with 50 nM PMA for 48 h, washed, resuspended in RPMI1640 containing 0.1% BSA in the culture dish, and recovered. The suspended cells were fixed with 4% paraformaldehyde solution, stained with either Hpse-Fc or mAb 10E4 (0.85 µg/mL), followed by relevant secondary antibodies labeled with FITC. Alternatively, the PMA-treated cells were incubated in glass slides equipped with small wells (4~10 × 10^4^ cells in a chamber of 8 × 5 mm) [46] for 120 min, fixed with 4% paraformaldehyde solution, and stained with Hpse(mut)-Fc (0.85 µg/mL) or other cell marker antibodies as in the histochemical staining procedure. For double staining, binding of Hpse(mut)-Fc was detected with anti-muHpse mAb RIO-1, followed by Alexa 488-goat anti-rat IgG(H+L) antibody, while binding of anti-SDC1 mAb was detected with biotinylated goat anti-mouse IgG F(ab’)_2_ fragment, followed by Alexa 568-labeled streptavidin. Stained images were obtained using confocal microscopy (FV3000, Olympus, Tokyo, Japan).

### 4.9. Precipitation of Hpse(mut)-Fc Binding Molecules from Monocytic U937 Cells

The PMA-treated U937 cells (4 × 10^7^ cells) were cultured in RPMI1640 containing 0.1% BSA, collected, and lysed with lysis buffer (20 mM Tris-HCl (pH 7.6) containing 2.5% Triton X-100 and 100 µM PMSF). The lysates were initially mixed with protein A sepharose to remove non-specifically adsorbed proteins. The treated supernatant was collected and mixed with protein A sepharose pretreated with Hpse(mut)-Fc or with the anti-SDC1 antibody. After gentle mixing overnight, the resin was recovered, washed, treated with the sample buffer, and subjected to SDS-PAGE. In separate experiments, the binding to the anti-SDC1 antibody (D4Y7H) and Hpse(mut)-Fc was tested by Western blot.

The proteins associated with Hpse(mut)-Fc were identified by mass spectrometry. The protein bands resolved on a SDS-PAGE gel were excised and treated with 10 mM dithiothreitol and 55 mM iodoacetamide. The samples were then digested with trypsin and desalted with an SDB tip (GL Sciences, Tokyo, Japan) before mass spectrometry. Data were analyzed using the Mascot tandem mass spectrometry software (Matrix Science, London, UK; version 3.1.0).

### 4.10. Statistical Analysis

Significant differences in the data were evaluated using the two-tailed Student’s *t*-test. A *p*-value of less than 0.05 was considered statistically significant.

## Figures and Tables

**Table 1:** (**a**) Hpse(mut)-Fc immunoprecipitated samples: band A ^1^. (**b**) Hpse(mut)-Fc immunoprecipitated samples: band B ^1^. (**c**) Hpse(mut)-Fc immunoprecipitated samples: band C ^1^. (**d**) Hpse(mut)-Fc immunoprecipitated samples: band D ^1^.

(a)	
Proteins	Total Spectrum Count
Spectrin beta chain, non-erythrocytic 1	2574
Spectrin alpha chain, non-erythrocytic 1	1971
Alpha-actinin-4	739
Filamin A	376
Myosin-9	356
Ras GTPase-activating-like protein IQGAP1	149
**(b)**	
**P** **roteins**	**Total Spectrum Count**
Alpha-actinin-4	1494
Spectrin alpha chain, non-erythrocytic 1	1254
Spectrin beta chain, non-erythrocytic 1	302
Actin, cytoplasmic 1	135
Ras GTPase-activating-like protein IQGAP1	61
**(c)**	
**P** **roteins**	**Total Spectrum Count**
Actin, cytoplasmic 1	1745
Alpha-actinin-4	95
Eukaryotic initiation factor 4A-III	29
Tubulin alpha-1B chain	27
Eukaryotic initiation factor 4A-I	19
**(d)**	
**P** **roteins**	**Total Spectrum Count**
Actin, cytoplasmic 1	831
Alpha-actinin-4	74
Lymphocyte-specific protein 1	19
Tubulin beta chain	12
Twinfilin-2	11

^1^ The detected bands in CBB staining, shown in Figure 6, were submitted to mass spectrometric identification. The bands A~D correspond to the band over 180 kDa (band A), 130 kDa (band B), 44 kDa (band C), and 39 kDa (band D), respectively.

## Data Availability

The original contributions presented in this study are included in the article. Further inquiries can be directed to the corresponding author.

## Supplementary Materials

The following supporting information can be downloaded at https://www.mdpi.com/article/10.3390/ijms262311293/s1.

## Abbreviations

CBB	Coomassie Brilliant Blue
CS	chondroitin sulfate
ELISA	enzyme-linked immunosorbent assay
GAG	glycosaminoglycan
HA	hyaluronic acid
HBD	heparin binding domain
Hpse	heparanase
L-Hpse	recombinant mouse Hpse protein mimicking latent-form Hpse
M-Hpse	recombinant mouse Hpse protein mimicking mature-form Hpse
Hpse-Fc	chimeric molecule of mouse heparanase and Fc region of mouse IgG1
HRP	horseradish peroxidase
HS	heparan sulfate
mAb	monoclonal antibody
MFI	median fluorescence intensity
mut	mutated (with mutation at E335A)
PMA	phorbol myristate acetate
SDC	syndecan
wt	wild type
